# Apoptotic Neutrophils Augment the Inflammatory Response to *Mycobacterium tuberculosis* Infection in Human Macrophages

**DOI:** 10.1371/journal.pone.0101514

**Published:** 2014-07-07

**Authors:** Henrik Andersson, Blanka Andersson, Daniel Eklund, Eyler Ngoh, Alexander Persson, Kristoffer Svensson, Maria Lerm, Robert Blomgran, Olle Stendahl

**Affiliations:** Division of Microbiology and Molecular Medicine, Department of Clinical and Experimental Medicine, Faculty of Health Sciences, Linköping University, Linköping, Sweden; Karolinska Institutet, Sweden

## Abstract

Macrophages in the lung are the primary cells being infected by *Mycobacterium tuberculosis* (Mtb) during the initial manifestation of tuberculosis. Since the adaptive immune response to Mtb is delayed, innate immune cells such as macrophages and neutrophils mount the early immune protection against this intracellular pathogen. Neutrophils are short-lived cells and removal of apoptotic cells by resident macrophages is a key event in the resolution of inflammation and tissue repair. Since anti-inflammatory activity is not compatible with effective immunity to intracellular pathogens, we therefore investigated how uptake of apoptotic neutrophils modulates the function of Mtb-activated human macrophages. We show that Mtb infection exerts a potent proinflammatory activation of human macrophages with enhanced gene activation and release of proinflammatory cytokines and that this response was augmented by apoptotic neutrophils. The enhanced macrophage response is linked to apoptotic neutrophil-driven activation of the NLRP3 inflammasome and subsequent IL-1β signalling. We also demonstrate that apoptotic neutrophils not only modulate the inflammatory response, but also enhance the capacity of infected macrophages to control intracellular growth of virulent Mtb. Taken together, these results suggest a novel role for apoptotic neutrophils in the modulation of the macrophage-dependent inflammatory response contributing to the early control of Mtb infection.

## Introduction

Resident alveolar and recruited macrophages in the lung are the primary cells being infected during tuberculosis. *Mycobacterium tuberculosis* (Mtb) has a unique ability to establish itself in these cells, primarily through its capacity to inhibit phagolysosome maturation and resist intracellular antimicrobial agents, such as reactive oxygen and nitrogen species (ROS, NO) [1], [2]. Even if T-cell mediated CD4+/CD8+ response eventually participates in the control of the infection, the onset of a CD4+ response against Mtb is slow. Therefore, innate immune cells, such as neutrophils and peripheral blood-derived macrophages, are recruited to the site of infection and constitute an early immune protection against this intracellular pathogen [3].

Neutrophils (PMNs) are short-lived cells that undergo apoptosis either spontaneously within 24 hours in circulation, or as a result of encountering invading pathogens [4]. Our understanding of the role of the PMNs in inflammation has expanded from their fundamental function as phagocytic cells that kill pathogens, to their capacity to regulate inflammatory responses [5]. Since apoptosis is an inevitable fate, apoptotic PMNs are recognized and cleared by professional phagocytes, including macrophages and dendritic cells (DCs) [6], [7]. During sterile conditions, this removal of apoptotic cells is immunologically neutral or results in an anti-inflammatory regulation and resolution of inflammation.

It is evident that uptake of apoptotic cells by macrophages in sterile models coincides with anti-inflammatory events such as down-regulation of proinflammatory cytokines, *e.g.* GM-CSF, IL-1β, IL-12 and TNFα and up-regulation of certain anti-inflammatory cytokines *e.g.* IL-10, TGF-β, PGE-2 and PAF [8]. Lack of TNFα and IFN-γ and abundance of anti-inflammatory cytokines facilitate intracellular growth and survival of Mtb [9], [10]. Thus, macrophage interaction with apoptotic cells during Mtb infections would be detrimental to the host, if it unconditionally leads to down-regulation of the inflammatory response. It is however believed that during the early phase of Mtb infection, PMNs can enhance host protection by secreting chemokines (IP-10, MCP-1) and cytokines such as IFN-γ and TNFα, which are crucial for the recruitment and activation of other immune cells [5]. PMNs also effectively engulf Mtb and kill them via ROS and anti-microbial peptides [11].

To date, most studies concerning resolution of inflammation by apoptotic cells describe the events in sterile models, thereby neglecting the most common factor for triggering inflammation; an infection. We have previously shown that PMNs, undergoing apoptosis as a result of ingesting Mtb, elicits a proinflammatory response in macrophages by releasing neutrophil extracellular traps (NETs) and Hsp72 [12]–[14]. In addition, these apoptotic cells have the capacity to induce maturation of DC [15]. This led us to further investigate the effects of clearance of spontaneously apoptotic PMNs by Mtb-infected macrophages. We now show that apoptotic PMNs (PMNapo) modulate the cytokine response of Mtb-infected macrophages through a caspase-1- and IL-1β-dependent process. Activation of macrophages included enhanced gene expression and release of proinflammatory cytokines, as well as enhanced capacity to control intracellular growth of virulent Mtb. Thus, PMNapo provide an early stimulation for Mtb-infected and anergic macrophages.

## Materials and Methods

### Reagents

TACS Annexin V-FITC was obtained from R&D Systems (McKinley Place, MN), DMEM and RPMI cell culture media, penicillin-streptomycin (PEST), gentamicin, L-glutamine and fetal bovine serum (FBS) were obtained from Gibco (Grand Island, NY), BD-Cytometric Bead Array Human Inflammation Kit, GolgiStop, CytoFix/CytoPerm, PermWash and Middlebrook 7H9 broth was obtained from BD Biosciences (San Diego, CA, USA), cell isolation components Polymorphprep and Lymphoprep were purchased from Axis-Shield (Oslo, Norway), heparin was obtained from LEO Pharma (Malmö, Sweden), latex microspheres (4.6 µm) were obtained from Polysciences Inc. (Warrington, PA). Cytochalasin D was purchased from Calbiochem (La Jolla, CA). PKH26 Red fluorescent cell linker minikit, Triton-X 100 and staurosporine (*Streptomyces sp.*) were purchased from Sigma Aldrich (Saint Louis, MO), Ac-YVAD-CMK was purchased from Cayman Chemical (MI, USA), and *M. tuberculosis* H37Rv (γ-radiation inactivated) was kindly provided by Colorado State University. Antibodies for intracellular cytokines were purchased from BioLegend (San Diego, CA, USA). Trizol Reagent, Superscript II and Purelink PCR Purification Kit were obtained from Invitrogen (Grand Island, NY). Turbo DNA-free kit was purchased from Ambion (Austin, TX) and SYBR Green PCR Master Mix from Applied Biosystems (Foster City, CA). Heparinized peripheral blood, buffy coats and normal human serum (NHS) were obtained from the blood bank at Linköping University Hospital (Linköping, Sweden), KRG (Krebs-Ringer phosphate buffer) containing 120 mM Na_2_HPO_4_ and 10 mM glucose with or without 1 mM CaCl_2_, PBS containing 137 mM NaCl, 2.7 mM KCl, 6.7 mM Na_2_PHO_4_, 1.5 mM KH_2_PO_4_ (pH 7.3) were prepared in-house.

### Monocyte separation and culture

Buffy coats were diluted 1∶1 with 0.9% NaCl and monocytes were separated over a Lymphoprep gradient, followed by collection in PBS with heparin (5000 IE/ml) and multiple washes in KRG, or derived directly from whole blood using a Lymphoprep gradient. Purified cells were resuspended in DMEM containing PEST (100 µg/ml penicillin and 100 µg/ml streptomycin) and 2 mM L-glutamine, and were allowed to adhere in 75 cm^2^ flasks for 1 h at 37°C. Non-adhered cells were removed by multiple washings in KRG and monocyte derived macrophages (hMDM) were obtained by culturing for 6–8 days in DMEM containing 10% NHS at 37°C in 5% CO_2_, with media changed every second to third day for the duration of incubation. The day before experiments, the hMDMs were trypsinized, counted and re-seeded in 96 well plates (1×10^5^ cells per well) for Mtb infection and intracellular (IC) survival assay, in 24 well plates (1×10^6^ cells per well) for cytokine analysis, and in 12 well plates (5×10^5^ cells per well) for western blot experiments. In all experiments, except for IC survival assay, the medium was changed to DMEM supplemented with PEST and L-glutamine 1 h before stimulation.

### PMN separation and induction of apoptosis

Peripheral blood was separated over a Lymphoprep and Polymorphprep gradient through centrifugation, followed by collection of the PMNs in PBS. Red blood cells were lysed by hypotonic shock and PMNs were washed in KRG and resuspended in RPMI containing 2 mM L-glutamine and 10% heat-inactivated FBS. Cells were allowed to enter apoptosis by incubation in Eppendorf tubes for 18 hrs at 37°C. PMNapo were washed once and resuspended in appropriate medium before being exposed to the hMDMs.

### Evaluation of apoptosis in PMNs

PMNs were stained with FITC-Annexin V according to manufacturer's protocol to detect early apoptosis and counter-stained with propidium iodide to detect necrotic cells or cells with post-apoptotic features. Detection of stained cells was performed with flow cytometry using CELLQuest software (FACS-Calibur, BD Biosciences). 75–85% of isolated PMNs stained positive for the early apoptosis marker phosphatidyl serine, whereas <5% were detected as necrotic (data not shown) after 18 hours.

### Preparation of *M. tuberculosis*

For use in hMDM activation studies, frozen aliquots of γ-radiation inactivated Mtb H37Rv (γ-irr Mtb) were thawed, resuspended in KRG with 0.1% Tween-20, centrifuged at 3000×g, resuspended in KRG with 0.1% Tween-20, passed multiple times through a 27-gauge syringe to ensure adequate separation of the bacteria and opsonized for 30 min in 50% NHS at 37°C. After opsonization, the bacteria were pelleted at 3000×g and resuspended in DMEM supplemented with PEST, L-glutamine and 1% NHS. For use in an intracellular survival assay, the virulent Mtb H37Rv strain harboring pSMT1-plasmid for luciferase-expression was kindly provided by Dr Kris Huygen [16]. The bacteria were grown in Middlebrook 7H9 broth with Tween-80, 0.5% glycerol and 10% albumin, dextrose and catalase for two to three weeks at 37°C, and then passaged and incubated for 7 days to early log phase, before being used.

### Macrophage stimulation

Cultured hMDMs were stimulated for 1 h with either γ-irr Mtb (5∶1 or 10∶1), PMNapo (2∶1) or 4.6 µm latex microspheres (2∶1). Although these MOI resulted in a low percentage of infected macrophages (Fig S2 and Table 1), these MOI were chosen for two reasons. The low number of infected macrophages reflects the *in vivo* situation, and higher MOI causes increased macrophage cell death. Non-ingested cells and bacteria were removed by multiple washings, and the culture media was centrifuged at 10,000×g to remove residual bacteria and particulate debris, and was then re-added to respective cell culture for an additional 18–20 h at 37°C before supernatants were collected and analyzed for cytokine content. To evaluate the interactions needed for PMNapo to exert synergistic activation, hMDMs were stimulated with the two stimuli together or separately with multiple washings between. To inhibit F-actin mobilization and consequently phagocytic uptake, the hMDMs were pre-incubated with 10 µM cytochalasin D (CytD) one hour before stimulation. To establish the role of caspase-1 in the activation of hMDMs, cells were pre-incubated with 50 µM Ac-YVAD-CMK (caspase-1 inhibitor) one hour prior to stimulation with γ-irr Mtb and/or PMNapo.

**Table 1:** Uptake of Mtb and PMNapo.

% ± SEM	No prey	Mtb	PMNapo	Mtb (5∶1)
		(5∶1)	(2∶1)	+ PMNapo (2∶1)
Neg (FITC^−^ PKH26^−^)	99±0,1	95±0,6	63±5,5	57±6,0
Mtb (FITC^+^ PKH26^−^)	0	4,2±0,7	0	2,5±0,6
PMNapo (FITC^−^ PKH26^+^)	0	0	36±5,6	39±6,7
Mtb+PMNapo (FITC^+^ PKH26^+^)	0	0	0	1,0±0,1

hMDMs were stimulated with FITC labeled γ-irr Mtb with or without subsequent stimulation with PKH26-labeled PMNapo. The uptake of Mtb and PMNapo was analyzed by flow cytometry. Values represent percentage of positive cells ± SEM (n = 5).

### Gene expression analysis

RNA isolation was performed using Trizol Reagent and to reduce possible DNA contamination the RNA was treated with DNase according to the manufacturer's protocol. The RNA was transcribed to cDNA using Superscript II according to manufacturer's protocol and the cDNA was purified using the Purelink PCR Purification Kit. Specific primers for selected genes were designed using the Primer3 program (http://frodo.wi.mit.edu/primer3/). Quantitative real-time PCR was performed in a 7900HT Fast Real-Time PCR. One reaction contained 12.5 µl SYBR Green PCR Master Mix, 1 µl of each primer (final concentration 800 nM) and 5 µl of cDNA. Total volume was adjusted with double distilled H_2_O to 25 µl. The housekeeping gene β-actin was used for normalization. The changes in gene expression are presented as ratios between hMDMs treated with stimuli and hMDMs without treatment (NC).

To investigate possible contribution of real-time PCR results from residual RNA coming from apoptotic neutrophils, RNA yield and expression of β-actin and TNFα in PMNapo was measured. The amount of RNA extracted from PMNapo was 10% of that isolated from hMDMs. Real-time PCR detected expression of both β-actin and TNFα in RNA from PMNapo, however the expression level in PMNapo compared to hMDMs was <<0.1% indicating that most of the RNA extracted was not intact. This shows that the results reflect gene expression in hMDMs only, with no major contribution from PMNapo.

### Cytokine analysis

Cytokine profiles in the culture supernatants were determined by cytometric bead array analysis, performed according to the manufacturer's protocol (BD Biosciences). Data and cytokine concentrations were analyzed using the Kaluza software (Beckman Coulter, Fullerton, CA, USA) and Microsoft Excel.

### Intracellular survival of Mtb

Intracellular survival of Mtb H37Rv was investigated as previously described [17]. Briefly, hMDMs were infected with Mtb, containing a pSMT1-plasmid for luciferase-expression, at a ratio of 10 Mtb per hMDM for 1 h at 37°C before hMDM were washed to remove extracellular bacteria, and medium was changed to serum-containing, antibiotic-free DMEM. Following infection hMDMs were stimulated with PMNapo at a ratio of two PMNapo per hMDM. At day 0 (4 hours after addition of PMNapo), 5 and 7 the cell culture supernatants were collected and hMDMs were lysed with water and luciferase expressing Mtb in cell culture supernatants and cell lysates were detected by addition of substrate (1% decanal) before luminescence was measured using a Modulus Microplate Multimode Reader equipped with an injector (Turner Biosystems, Sunnyvale, CA, USA). Data was obtained as arbitrary luminescence units (ALU).

### Quantification of uptake

γ-irr Mtb was prepared as above, with the following addition: Prior to opsonisation the bacteria were labelled with FITC (0.1 mg/ml) for 30 min at 37°C and then washed once in KRG with 0.1% Tween-20. PMN were labeled with PKH26 and incubated for induction of apoptosis. hMDM stimulation with labeled Mtb and PMNapo was performed as above. hMDMs were harvested by trypsinization, washed thoroughly in PBS and fluorescence was measured on a Gallios flow cytometer (Beckman Coulter). Data was analyzed using Kaluza software (Beckman Coulter), where hMDMs that ingested non-stained PMNapo and Mtb served as background for quantifying the amount of cells that were positive for PKH26-labeled PMNapo, FITC-labeled Mtb or both.

### Staining for intracellular cytokines

Cells were stimulated as described above for 2 h, before brefeldin A (GolgiStop, BD Biosciences) was added, to block TNFα secretion, for an additional 2 h. Cells were harvested by trypsinization and washed thoroughly in PBS before addition of Cytofix/Cytoperm for 20 min at 4 °C. This was followed by additional washing steps with PermWash and staining with Pacific Blue-conjugated anti-human IL-1β, Alexa 647-conjugated anti-human TNFα or the corresponding isotype control for 30 min at room temperature. Finally, cells were washed and fluorescence was measured on a Gallios flow cytometer (Beckman Coulter). Data was analyzed using Kaluza software (Beckman Coulter), where hMDMs that ingested non-stained PMNapo and γ-irr Mtb served as background for quantifying the amount of cells that were positive for PMNapo, Mtb or both. After gating on these 4 subpopulations (FITC^−^PKH26^−^ (no uptake); FITC^−^PKH26^+^ (PMNapo); FITC^+^PKH26^−^ (Mtb); FITC^+^PKH26^+^ (Mtb+PMNapo)), cells positive for TNFα (FL-6) or IL-1β (FL-9) could be determined, with the appropriate isotype controls serving as background fluorescence.

### Caspase-1 activity by western blotting

At the indicated time point, hMDMs in 12-well plates where washed with PBS and immediately lysed by adding 300 µl boiling Laemmli sample buffer and additionally boiled for 10 min. Samples were treated with benzonase (Merck) for 30 min at 37°C and equal amounts of lysates were separated by 8–16% SDS polyacrylamide gel electrophoresis (PAGE) and electrotransferred onto PVDF membranes. The membranes were blocked with 5% milk powder in PBS Tween-20 (0.075%) and incubated with a primary antibody recognizing the p45 proenzyme (∼45 kDa) and the p20 subunit (∼20 kDa) of caspase-1 (1∶1000; Merck), followed by stripping and re-probing with β-actin (1∶10 000) as a loading control. The specific proteins were detected with a commercial enhanced luminol-based chemiluminescent (ECL) kit (Amersham). Densitometric analysis of bands was performed using NIH/ImageJ, and a caspase-1 p20/p45 ratio (cleaved active caspase-1/unprocessed inactive caspase-1) was constructed to evaluate caspase-1 activation in a way that was unbiased of difference in total protein across samples.

### Statistical analysis of data

All data are presented as mean + SEM. Statistical significance and differences between groups were calculated using Student's t-test and one-way ANOVA, using the software Prism 5 (Graphpad application). Significance levels at p<0.05 are depicted as *, p<0.01 as ** and p<0.001 as ***.

## Results

### Apoptotic neutrophils augment activation of Mtb-infected hMDMs

Uptake of PMNapo *per se* does not trigger a proinflammatory activation of macrophages in sterile models; it is rather the opposite as apoptotic cells promote anti-inflammatory signaling and resolution of inflammation [8]. Abundance of apoptotic debris at the inflammatory site may therefore challenge the inflammatory capability of macrophages. To understand the role of apoptotic cells during intracellular Mtb infections, we explored the immunomodulatory effects of PMNapo on human macrophages, infected with Mtb H37Rv.

**Figure 1:**
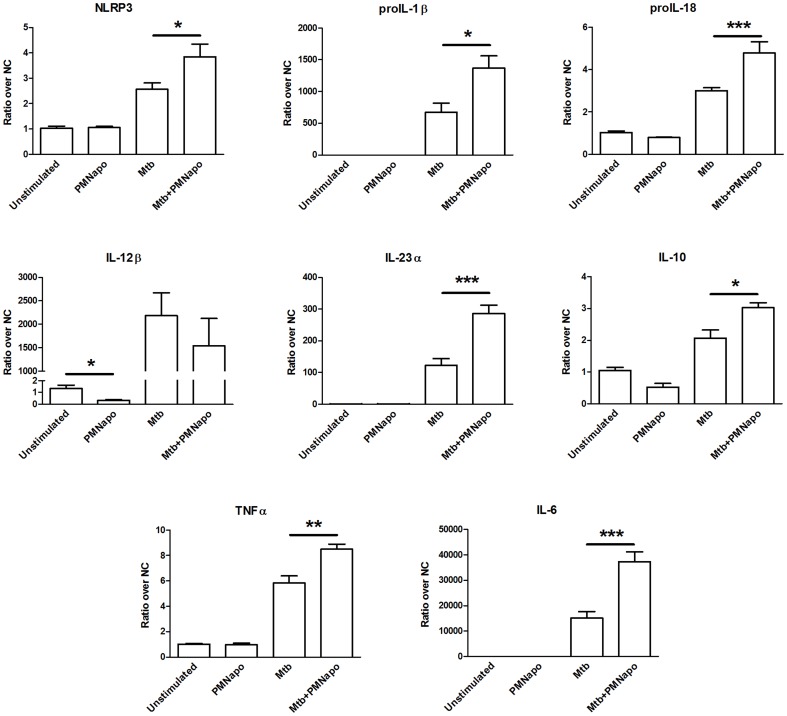
Apoptotic neutrophils augment the inflammatory response of macrophages to Mtb. hMDMs were stimulated with γ-irr Mtb at a ratio of 5∶1 (Mtb) for one hour, with or without subsequent stimulation with PMNapo at a ratio of 2∶1 (Mtb+PMNapo) for one hour, where after the hMDMs were re-cultured for 20 h. hMDMs stimulated with PMNapo without prior stimulation with Mtb (PMNapo) was included as control. mRNA levels were measured by quantitative real-time PCR. Data are presented as ratios over untreated cells (NC) + SEM (n = 7). Differences between groups are shown as * (p<0.05), ** (p<0.01) or *** (p<0.001).

hMDMs were infected with γ-irr Mtb (MOI 5∶1), with or without subsequent exposure to PMNapo, and incubated for 20 h. We examined the gene expression of several pro- and anti-inflammatory cytokines as well as innate immune receptors (TLR2/4, NLRP3). γ-irr Mtb increased expression of all proinflammatory cytokines analyzed (proIL-1β, proIL-18, IL-12β, IL-23α, IL-6, TNFα), as well as IL-10 and NLRP3 (Fig. 1), TLR2 and TLR4 (data not shown). Addition of PMNapo alone did not change the basal levels of these genes in non-infected hMDMs, except for IL-12β, which was 3-fold downregulated. When PMNapo were added to γ-irr Mtb-infected hMDMs the expression of genes related to the NLRP3 inflammasome, namely NLRP3, proIL-1β and proIL-18, were significantly increased in Mtb- infected hMDMs. We also found a significant upregulation of the proinflammatory cytokines TNFα, IL-6 and IL-23α, while the expression of IL-12β was unchanged. Expression of the anti-inflammatory cytokine IL-10 was increased while TGFβ expression was unchanged. The upregulation of TLR4 by γ-irr Mtb was also augmented by subsequent addition of PMNapo. This led us to investigate if molecules downstream of IL-1R and TLRs were affected at a transcriptional level. However, the gene expressions of MyD88, IRAK4 or Tirap (data not shown) were not affected by stimulation with Mtb and/or PMNapo.

**Figure 2:**
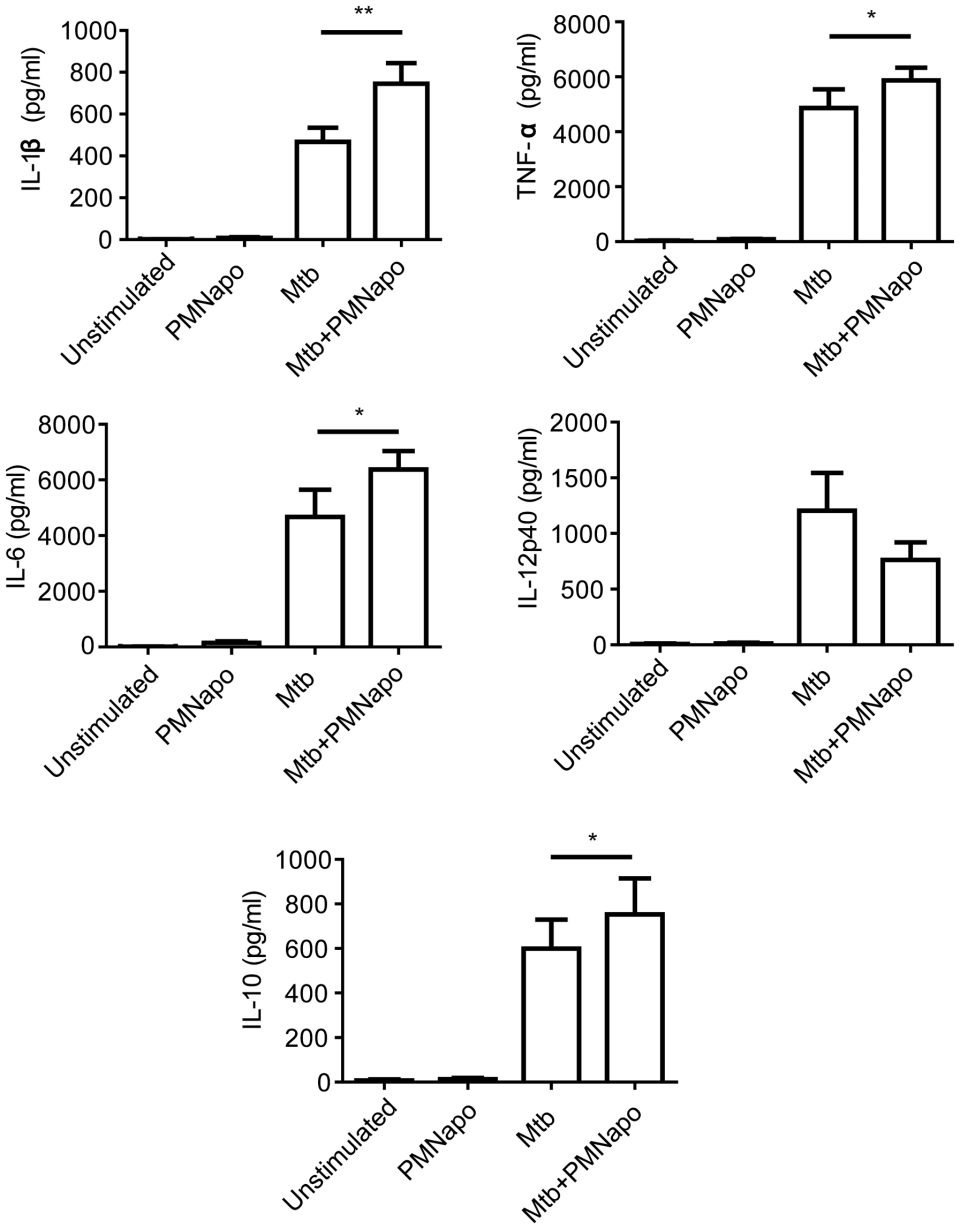
Apoptotic neutrophils augment the inflammatory response of hMDMs to Mtb. hMDMs were stimulated with γ-irr Mtb at a ratio of 5∶1 (Mtb) with or without PMNapo at a ratio of 2∶1 (+PMNapo) for one hour, where after non-ingested bacteria and cells were removed and the hMDMs were re-cultured with the supernatants for 18 h. Cytokine (TNFα, IL-1β, IL-6, IL-10 and IL-12p40) levels were measured by cytometric bead array and data are expressed as mean cytokine released + SEM (n = 8). Differences between groups are shown as * (p<0.05) or ** (p<0.01) or *** (p<0.001).

Changes in gene expression do not always translate to protein expression and secretion. We therefore analyzed protein levels of selected cytokines in the cell culture medium. The effect of PMNapo on γ-irr Mtb-infected hMDMs on cytokine release showed similar changes as seen on gene expression, with a significant increased secretion of IL-1β, TNFα, IL-6 and IL-10, and, although not statistically significant, decreased levels of IL-12p40 (Fig. 2).

### Presence of apoptotic cells enhances the hMDM capacity to control intracellular Mtb

**Figure 3:**
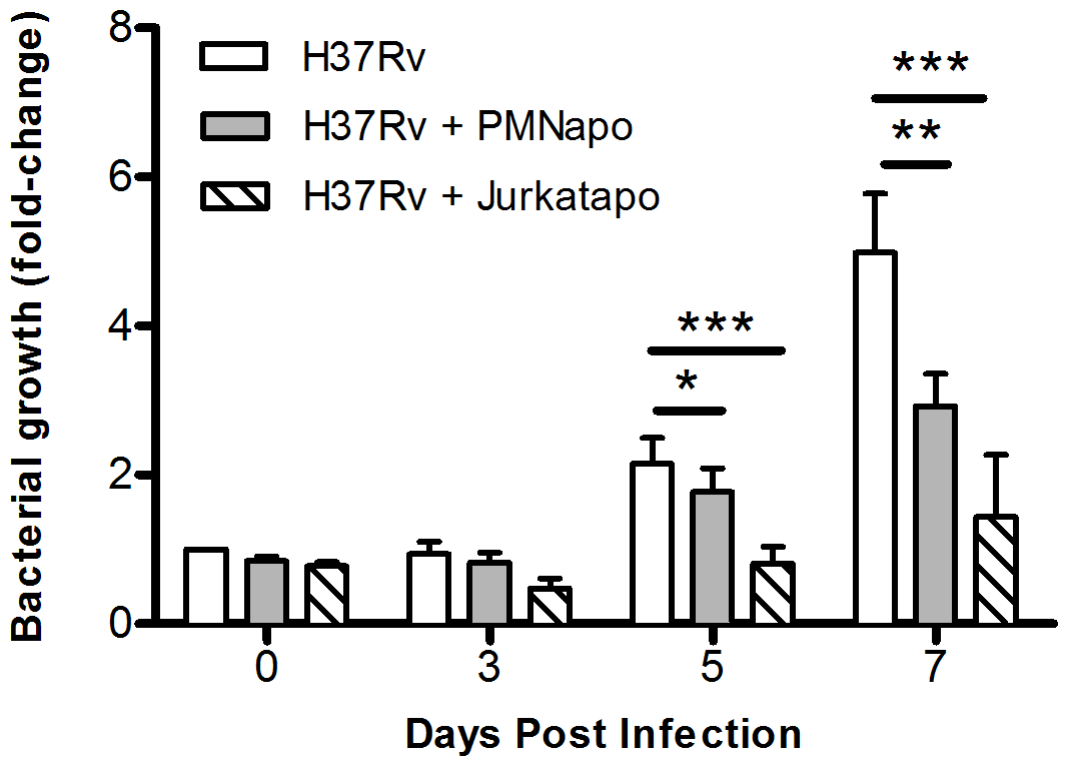
Apoptotic neutrophils enhance the hMDM capacity to control intracellular Mtb. hMDMs were infected with Mtb H37Rv and uptake was determined (D0). Following infection, hMDMs were stimulated with PMNapo or Jurkatapo at a ratio of 2∶1. Measurements of Mtb by luminometry were performed at indicated time points. Data are presented as ratio compared to the initial bacterial load at D0 (*i.e.* increase in bacterial load) and the graphs show the mean + SEM (n = 5). Differences between groups are shown as * (p<0.05), ** (p<0.01) or *** (p<0.001).

To show how PMNapo affect the capacity of the hMDMs to control intracellular growth of Mtb, we employed a recently developed human *in vitro* model for Mtb infection based on hMDMs infected with luciferase-expressing Mtb H37Rv [17]. We found that presence of PMNapo significantly improved the capacity of Mtb-infected hMDMs to restrict bacterial growth (Fig. 3). To investigate if this effect was specific for apoptotic PMN, we induced apoptosis in Jurkat T-cells (Jurkatapo) using 1 µg/ml staurosporine for two hours (resulting in 62% Annexin V+, with minor degree of Annexin V+PI+ cells, 5.4%), and presented them to Mtb-infected hMDMs. The restriction of bacterial growth was observed also for Jurkatapo.

### Phagocytic uptake of apoptotic neutrophils is required for hMDM activation

**Figure 4:**
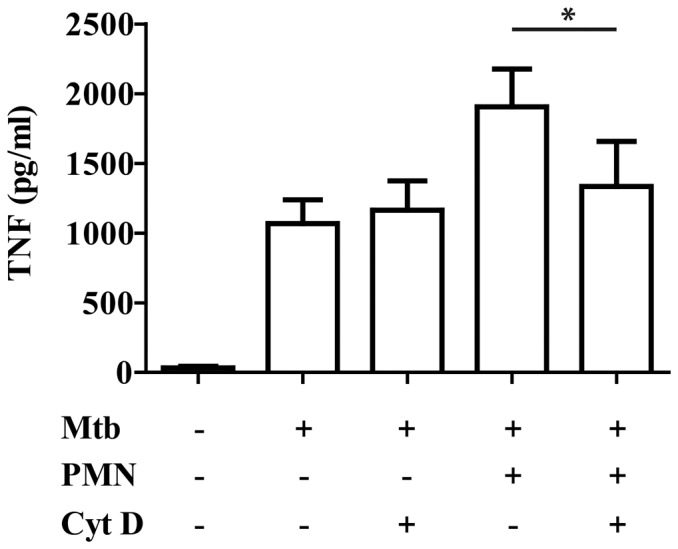
The augmentation of hMDM activation is dependent specifically on phagocytosis of apoptotic neutrophils. hMDMs were pre-incubated with or without cytochalasin D (CytD) prior to stimulation with γ-irr Mtb at a ratio of 5∶1 alone (Mtb) or together with PMNapo at a ratio of 2∶1 (Mtb + PMNapo) for one hour where after non-ingested prey were removed and the hMDMs were cultured for 18 h. Data are expressed as mean TNFα released + SEM (n = 3).

To evaluate if the PMNapo-induced augmentation requires efferocytosis by hMDMs, we inhibited the process with cytochalasin D (CytD). CytD treatment did not affect Mtb stimulation of hMDMs *per se*, but reduced the augmenting effect of PMNapo (Fig. 4), suggesting that enhanced activation of the hMDMs required phagocytosis of apoptotic cells or bodies. It could be argued that phagocytosis of a large prey *per se*, together with a bacterial stimulus affected the activation of the macrophage. We therefore allowed hMDMs to phagocytose an inert prey, latex beads, together with γ-irr Mtb. Phagocytosis of latex beads did not affect the proinflammatory activation of Mtb-infected hMDMs (Fig S1).

### Production of IL-1β and TNFα in hMDMs containing Mtb and/or PMNapo

When evaluating the number of hMDMs phagocytosing γ-irr Mtb and/or PMNapo, 2.5–4.2% of the hMDMs ingested Mtb, 36–40% ingested apoptotic cells, and only 1% ingested both Mtb and PMNapo (Table 1). The MOI had to be increased significantly to elevate the proportion of cells infected with γ-irr Mtb (Fig S2). This also resulted in high bacterial load per cell (data not shown), not reflecting the *in vivo* situation during the early phase of infection. Furthermore, higher bacterial load lead to significant cell death of infected hMDMs [18].

**Figure 5:**
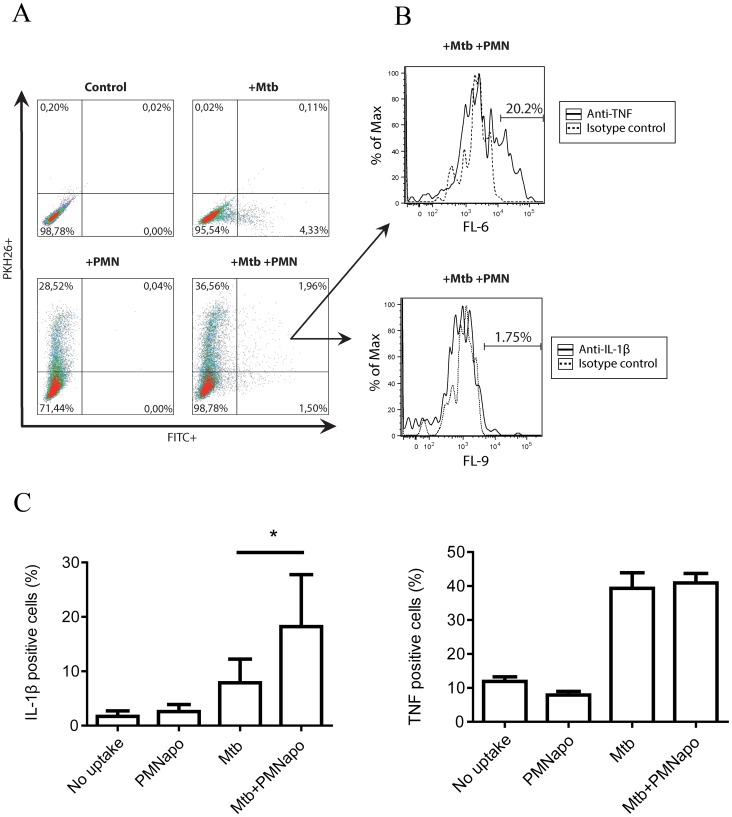
Uptake of apoptotic neutrophils increases the number of IL-1β producing cells in the infected cell population. hMDMs were stimulated with FITC labeled γ-irr Mtb at a ratio of 5∶1 (Mtb), with subsequent stimulation with PKH26-labeled PMNapo at a ratio of 2∶1 (Mtb+PMNapo). The hMDMs were stained for intracellular IL-1β (Pacific Blue) and TNFα (Alexa Fluor 647) and analyzed by flow cytometry. (A) The figure shows shows the percentage of hMDMs (based on forward/side scatter) positive for Mtb (FITC/FL-1), PMNapo (PKH26/FL-2) or both. (B) The histograms show the percentage of TNFα (Alexa 647/FL-6) or IL-1β (Pacific Blue/FL-9) positive cells in the population of hMDMs which have phagocytosed both Mtb and PMNapo. (C) The figure shows the number of IL-1β or TNFα producing cells (%) in the following subpopulations; FITC^−^/PKH26^−^ (no uptake), FITC^−^/PKH26^+^ (PMNapo uptake), FITC^+^/PKH26^−^ (Mtb uptake) and FITC^+^/PKH26^+^ (Mtb+PMNapo). (n = 5). Differences between groups are shown as * (p<0.05).

Staining for intracellular cytokines (IL-1β and TNFα) revealed that in the population staining positive for uptake of both PMNapo and γ-irr Mtb, the number of IL-1β-expressing cells was higher (28%), compared to those cells containing Mtb alone (12%) (Fig. 5). There was no difference between the number of TNFα producing cells in the population containing Mtb alone compared to cells harboring both Mtb and PMNapo (42%). When calculating the overall number of cytokine positive cells, given the low infection rate, it is evident that most of the cells expressing IL-1β or TNFα have not ingested Mtb and/or PMNapo, suggesting a paracrine stimulation of adjacent non-infected hMDMs, reflecting a potent activation of bystander cells.

### Apoptotic neutrophils augment hMDM activation through caspase-1 and release of IL-1β

**Figure 6:**
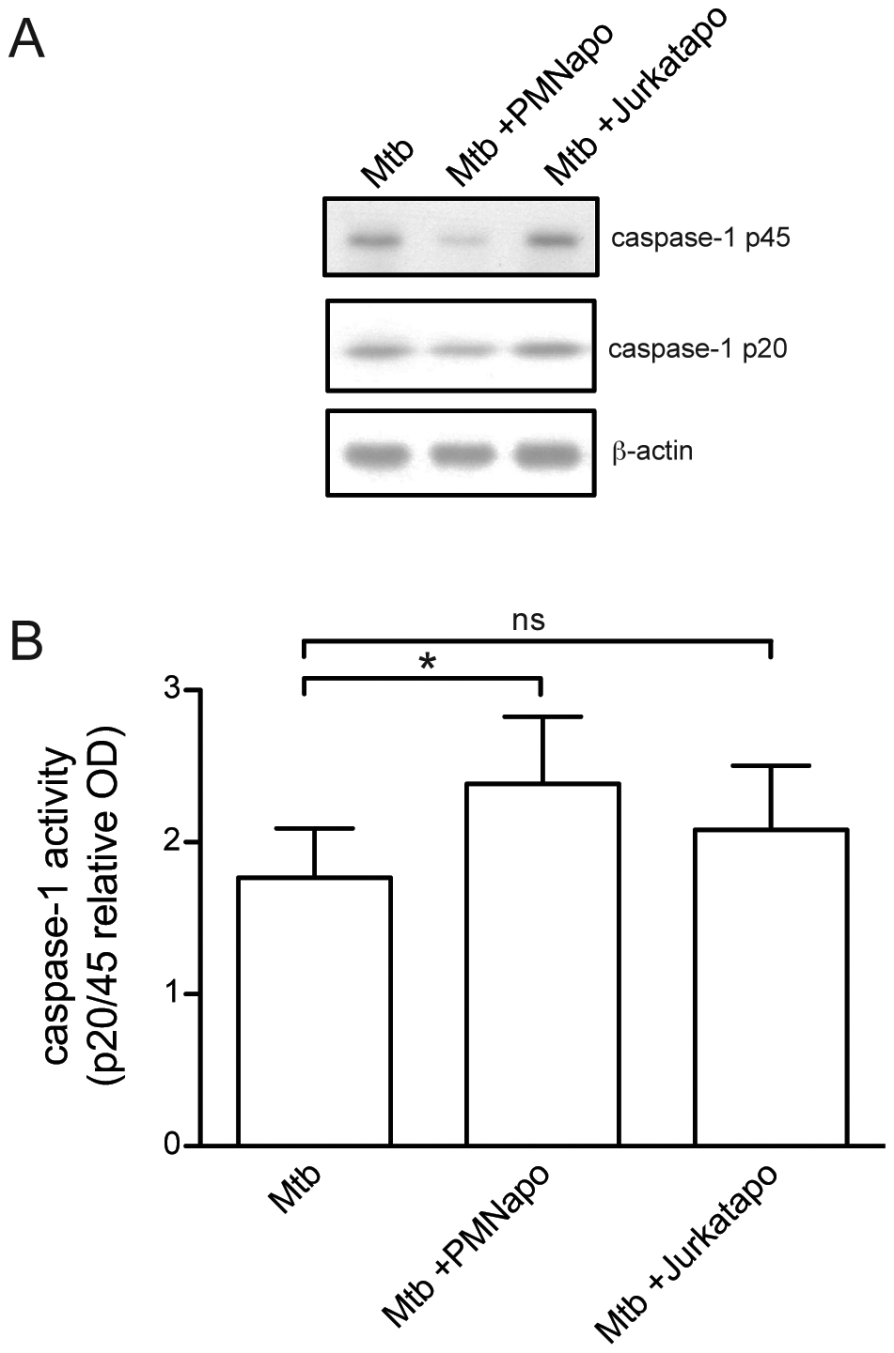
Apoptotic neutrophils augment caspase-1 activation in Mtb-stimulated hMDMs. hMDMs were stimulated with γ-irr Mtb for one hour with or without subsequent stimulation with PMNapo or Jurkatapo at a ratio of 2∶1 for one hour where after non-ingested prey were removed and the hMDMs were cultured for 3 h. hMDMs were extensively washed and lysed in Laemmli sample buffer. (A) Representative SDS-PAGE immunoblots of hMDM cell lysates probed with anti-caspase-1 antibodies, detecting both unprocessed inactive caspase-1 (p45) and cleaved activated caspase-1 (p20). Membranes were then stripped and re-probed for β-actin as a control for equal loading. (B) Caspase-1 activity shown as the ratio between caspase-1 p20/caspase-1 p45 and data expressed as mean + SEM (n = 4-5). Differences between groups were calculated using paired sample Student t-test, and significant differences are shown as * (p<0.05); ns, not significant.

Since PMNapo enhanced NLRP3, IL-1β and IL-18 expression, we focused on the putative role of intracellular innate immune recognition mechanisms via the NLRP3 inflammasome, since IL-1β has been shown to be involved in the innate immune response to Mtb [19]. This led us to investigate the role of caspase-1 activation and IL-1β release during hMDM activation. In order to evaluate differences in hMDM caspase-1 activation we employed western blotting making it possible to analyze both unprocessed caspase-1 (caspase-1 p45) and the cleaved active form (caspase-1 p20) (Fig. 6a). By forming a ratio between caspase-1 p20/caspase-1 p45 the relative caspase-1 activation could be compared between samples in an unbiased way where total protein had no influence (Fig. 6b). γ-irr Mtb-infected hMDMs stimulated with PMNapo showed approx. 1.4-fold increase in caspase-1 activation, compared to Mtb alone (p = 0.026). In an attempt to stimulate this augmenting effect on hMDM activation with another apoptotic prey, we induced apoptosis in Jurkat T-cells using 1 µg/ml staurosporine for four hours (resulting in 81% Annexin V+, with minor degree of Annexin V+PI+ cells, 4.6%), and presented them to Mtb-infected hMDMs. These apoptotic cells showed no significant augmenting effect on Mtb-infected hMDMs (p = 0.19). Furthermore, neither PMNapo nor Jurkatapo contributed with any measurable levels of caspase-1 protein (data not shown).

**Figure 7:**
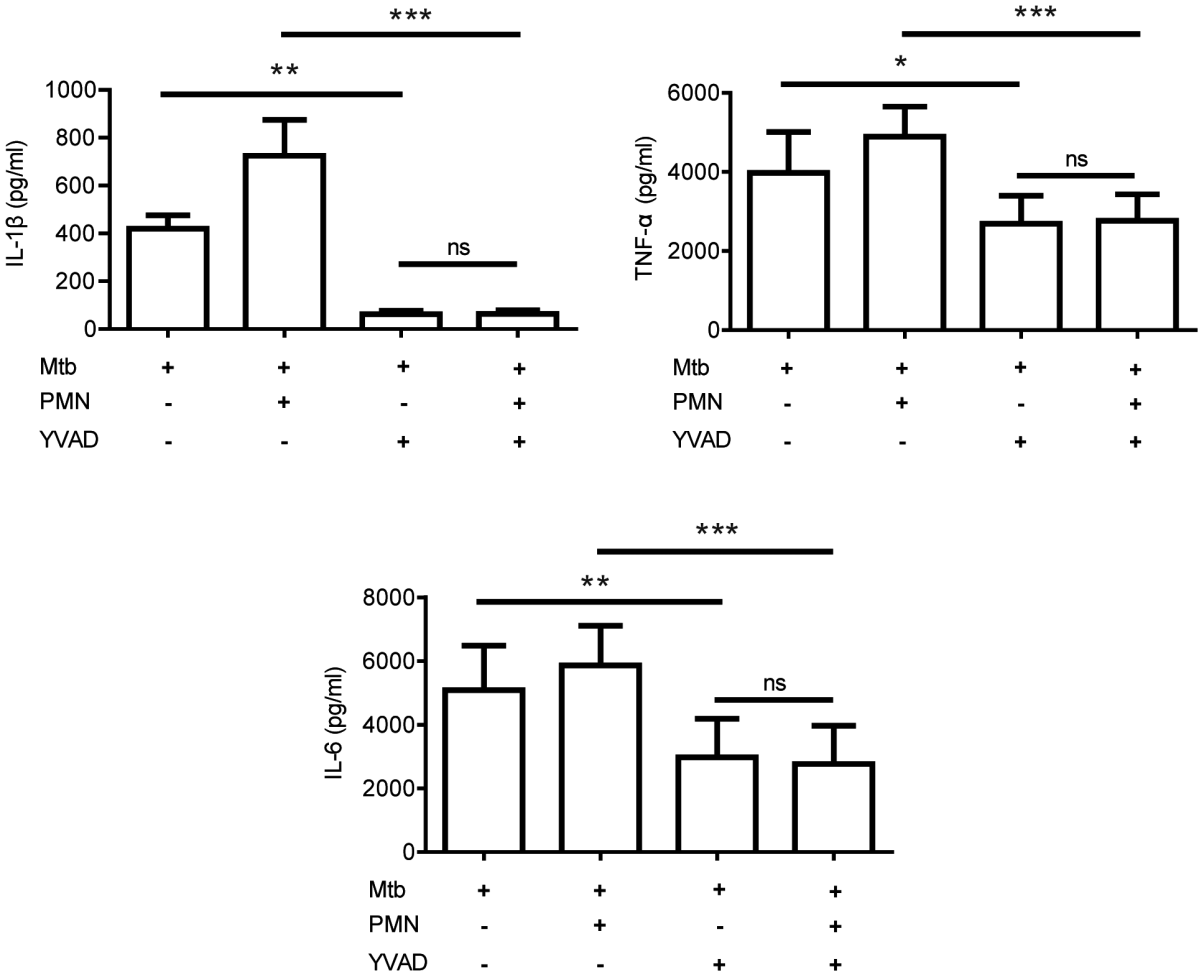
Apoptotic neutrophils augmentation of cytokine release depends on caspase-1 activity. hMDMs, with or without pre-incubation with Ac-YVAD-CMK (caspase-1 inhibitor), were stimulated with γ-irr Mtb with or without PMNapo at a ratio of 2∶1 for one hour where after non-ingested prey were removed and the hMDMs were cultured for 18 h. Cytokine levels were measured by cytometric bead array, and data are expressed as mean cytokine released + SEM (n = 6). Differences between groups are shown as * (p<0.05), ** (p<0.01) or *** (p<0.001).

To study the effect of caspase-1 activation on cytokine production we pre-incubated hMDMs with the caspase-1 inhibitor Ac-YVAD-CMK prior to stimulation with γ-irr Mtb/PMNapo. IL-1β levels decreased significantly in the presence of the inhibitor (Fig. 7). In addition, inhibition of caspase-1 led to a significant reduction of both TNFα and IL-6. The effect of caspase-1 inhibition on cytokine secretion from hMDMs stimulated with γ-irr Mtb only demonstrates the importance of the NLRP3 inflammasome in response to Mtb. The effect of the inhibitor on hMDMs stimulated with γ-irr Mtb and PMNapo was even greater, completely removing the augmenting effect of PMNapo. This suggests that PMNapo-driven caspase-1 activation and subsequent IL-1β release plays a pivotal role during the enhanced macrophage activation.

## Discussion

We have previously shown that Mtb-infected apoptotic PMNs can trigger a proinflammatory response in macrophages, mediated by the release of neutrophil extracellular traps (NETs) and heat-shock protein 72 from the Mtb-infected apoptotic PMNs [12]–[14]. Here we extend these observations, and show that despite having no direct effect on hMDM activation, non-infected apoptotic PMN can enhance hMDM response to Mtb. Our observation challenges earlier findings that apoptotic PMNs primarily impair the response to certain microbial stimuli [8], and that phagocytosis of apoptotic cells enhances intracellular survival of pathogens [20]–[22].

It could be anticipated that soluble stimuli from PMNapo or hMDMs could simultaneously activate both non-infected and infected hMDMs. Since both non-infected and infected hMDMs respond to PMNapo stimulation, a paracrine stimulation of surrounding hMDMs does occur. However, the fraction of IL-1β responding cells is higher in the hMDM population ingesting both Mtb and PMNapo, suggesting that the direct effect on the infected hMDMs is more effective. It could be argued that this population of infected hMDMs ingesting PMNapo is insignificantly small. However, in a latency model, where macrophages were able to control infection [18], and in the *in vivo* situation, only a small fraction of inflammatory cells are infected [23], suggesting that our *in vitro* model reflects the *in vivo* situation where both infected and adjacent non-infected hMDMs respond, and collectively contribute to Mtb control.

Our data show that the PMNapo-mediated inflammatory activation of Mtb-infected hMDMs resulted in high IL-10 levels, a characteristic of regulatory macrophages. These results confirm recent findings by Filardy *et al* [24], who showed that uptake of apoptotic neutrophils in the presence of a proinflammatory stimulus induces a regulatory phenotype with high IL-10 and low IL-12 in mouse macrophages. IL-10 has been shown to inhibit phagolysosome fusion [25] and NLRP3 activation [26], and IL-6 can inhibit IFN-γ induced autophagy [27]. Still, our observations suggest that the neutrophil-stimulated Mtb-infected hMDMs show a more M1-like phenotype [28], where proinflammatory cytokines, particularly IL-1β, can enhance the capacity of macrophages to control intracellular growth of pathogens. In fact IL-10 may be released from a subpopulation of non-infected macrophages.

Our experiments with luciferase-transfected Mtb H37Rv bacteria show that PMNapo can enhance hMDM control of intracellular growth of Mtb. One mechanism whereby PMNs may enhance macrophage microbicidal activity is by delivering granules containing microbicidal components to the Mtb-phagosome [29]. However, this restriction of bacterial growth was also observed using apoptotic Jurkat cells. These results show that the effect of apoptotic neutrophils can be mediated not only by transfer of neutrophil granules and antimicrobial peptides [29], but also by uptake of apoptotic cells affecting intracellular signaling and inflammasome activation. An alternative or complementary mechanism could be enhanced TNFα and IL-1β release affecting phagolysosomal maturation. It was recently shown that efferocytosis of apoptotic macrophages enhanced phagolysosome fusion and intracellular killing of Mtb [30]. Taken together, our results illustrate that the enhanced capacity of infected hMDMs to control the multiplication of intracellular Mtb in the presence of PMNapo is mediated via IL-1β. Although this effect was also observed using another apoptotic cell, in the early TB infection in the lung, the dominant cells are neutrophils and macrophages.

The fact that apoptotic PMNs must be ingested to modulate the response, suggests that certain intracellular recognition mechanisms must come into play. IL-1β plays a pivotal role in mediating host response to infection and danger molecules, and is released and secreted via inflammasome-dependent caspase-1 activation. NLRP3 is activated by Mtb virulence factors, such as ESAT-6, and host resistance to Mtb is highly dependent on IL-1β, which is found in the lavage fluid of TB patients [19], [31]. Enhanced gene activation of NLRP3 and the activation of caspase-1 show that the inflammasome is activated during Mtb- and PMNapo-mediated activation of hMDMs. Inhibition of caspase-1 did not only affect IL-1β release, but also TNFα and IL-6, suggesting that IL-1β plays a role in augmenting the proinflammatory response. It is tempting to speculate that when apoptotic PMNs or apoptotic bodies are internalized, they serve as a danger signal, which via the inflammasome activates caspase-1. This activation generates additional IL-1β and IL-18, which in turn acts as a feedback activator, and upregulates the production and release of other proinflammatory cytokines, such as TNFα and IL-6. The fact that caspase-1 inhibition not only inhibits release of IL-1β but also of TNFα and IL-6 supports this scenario. Furthermore, a recent study where infected macrophages were stimulated to release increased levels of IL-1β has shown that IL-1β exerts an antimicrobial effect by increasing the release of TNFα and upregulating the TNF receptor on infected cells, which in turn leads to increased apoptosis and efferocytosis of infected macrophages [32].

Anakinra, an anti IL-1β receptor antagonist, did however not reduce the antimicrobial activity in macrophages exposed to apototic neutrophils or Jurkat cells. One reason for this somewhat unexpected results, since we have previously observed increased bacterial growth in Anakinra-treated macrophages infected with low number of Mtb [33], could be that reduced IL-1β could augment other antimicrobial mechanisms, such as autophagy, as suggested by de Luca et al [34].

There are accumulating evidence showing that PMNs play an important role in the early phase of tuberculosis infection, both for antimicrobial activity, DC activation, antigen presentation and granuloma formation [15], [23], [29], [35]. Our results demonstrate that PMNapo have the ability to enhance the capacity of hMDMs to control the growth of intracellular bacteria. Whether this efferocytic process enhances phagolysosome fusion, autophagy, or delivery of microbicidal compounds needs further investigation.

## Supporting Information

Figure S1 The augmentation of hMDM activation by apoptotic neutrophils is not a result of simultaneous phagocytosis of additional prey. hMDMs were stimulated with medium (Medium), latex beads at a ratio of 5∶1 (Latex), γ-irr Mtb at a ratio of 5∶1 (Mtb) or Mtb and latex beads simultaneously (Latex+Mtb). Data are expressed as TNFα release normalized to hMDMs stimulated with Mtb alone which is indicated as 100%. Graph shows mean + SEM (n = 3).
(TIF)

Figure S2 The effect of increasing MOI on uptake of *M. tuberculosis*. hMDMs were stimulated with FITC labeled γ-irr Mtb at different ratios and analyzed by flow cytometry. Data are presented as percentage of hMDMs that had phagocytosed at least one bacterium (FITC^+^). Values represent mean + SEM (n = 4).
(TIF)
