# Supplementary figures and images for: Apoptotic Neutrophils Augment the Inflammatory Response to Mycobacterium tuberculosis Infection in Human Macrophages
Source: PLoS One. 2014 Jul 7;9(7):e101514. doi: 10.1371/journal.pone.0101514 (PMC4084802; doi:10.1371/journal.pone.0101514)

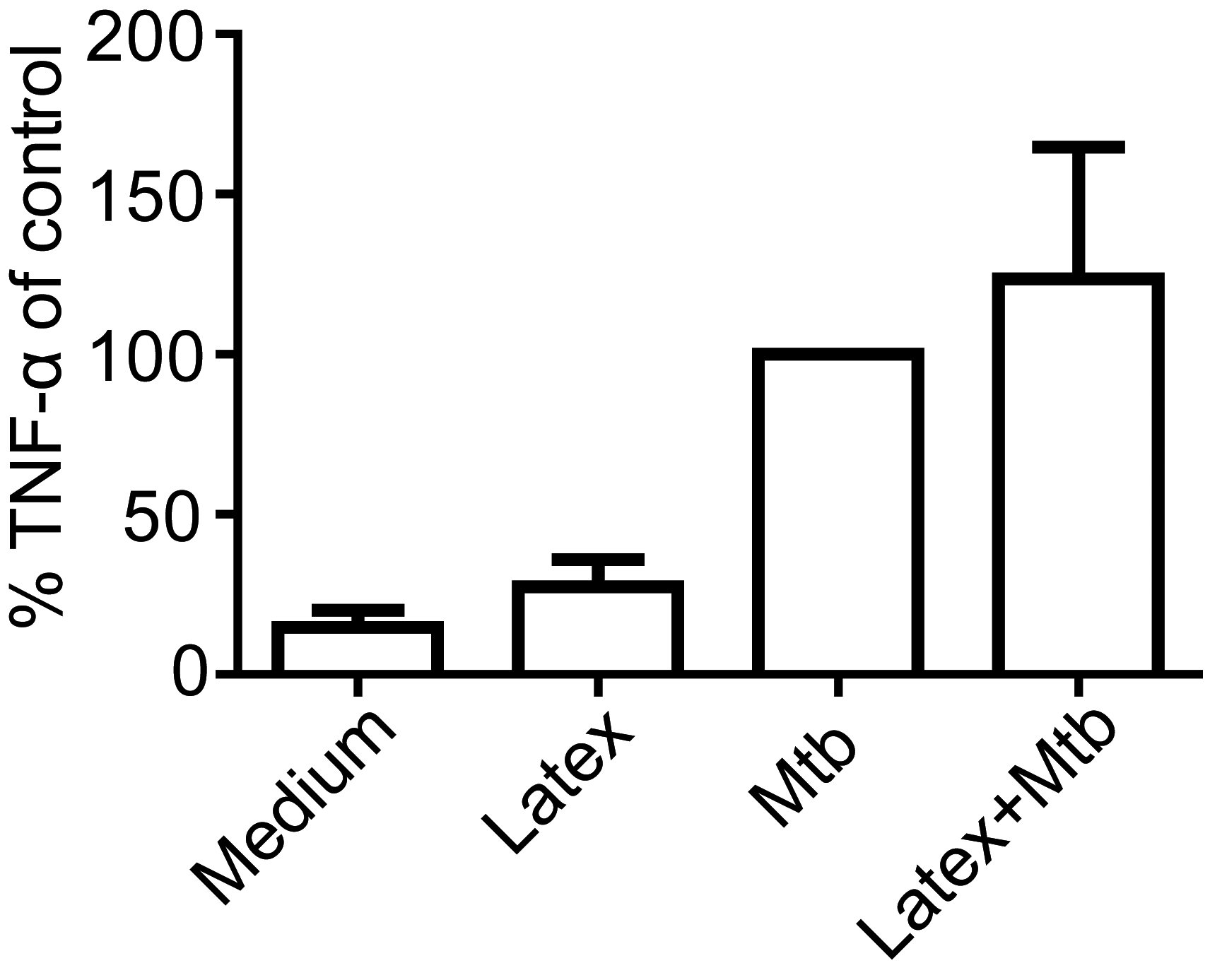

Supplement: Figure S1 — The augmentation of hMDM activation by apoptotic neutrophils is not a result of simultaneous phagocytosis of additional prey. hMDMs were stimulated with medium (Medium), latex beads at a ratio of 5∶1 (Latex), γ-irr Mtb at a ratio of 5∶1 (Mtb) or Mtb and latex beads simultaneously (Latex+Mtb). Data are expressed as TNFα release normalized to hMDMs stimulated with Mtb alone which is indicated as 100%. Graph shows mean + SEM (n = 3). (TIF) [file pone.0101514.s001.tif]

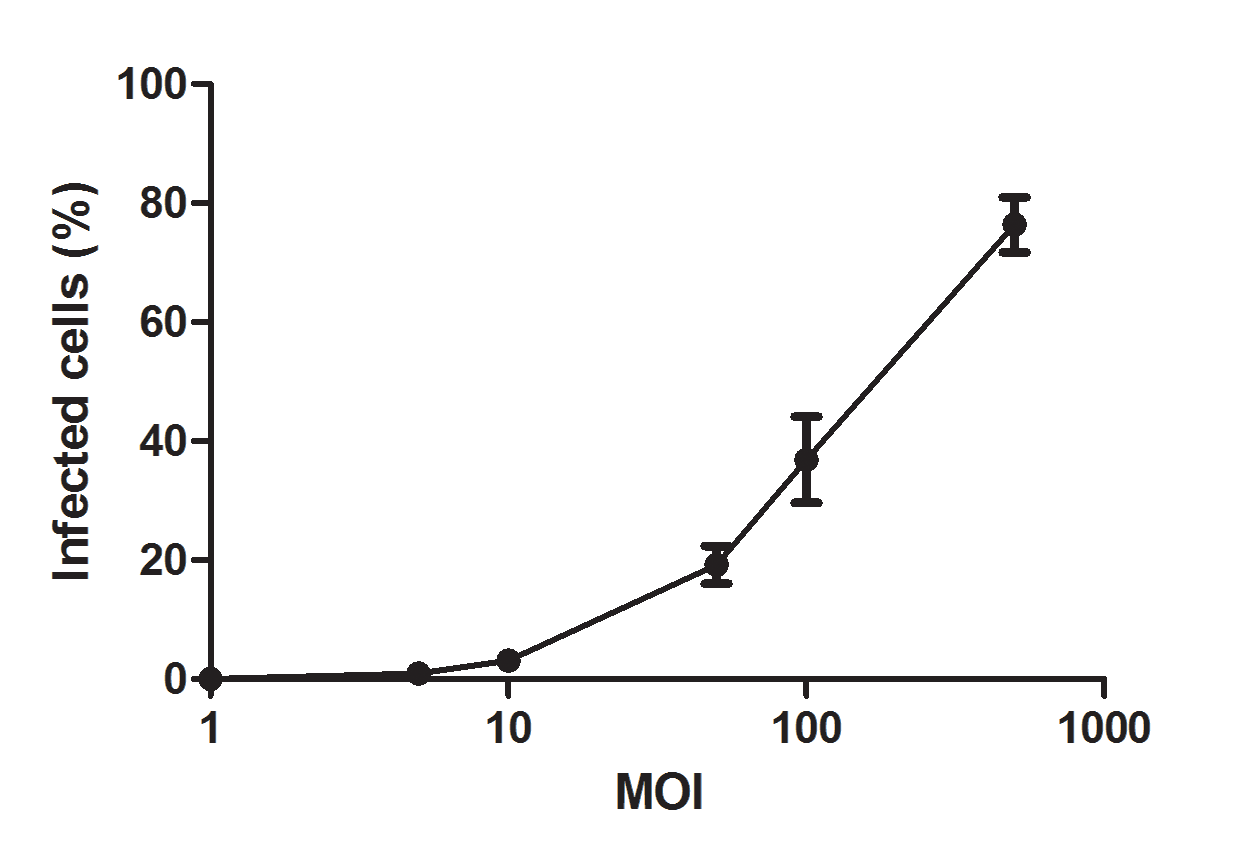

Supplement: Figure S2 — The effect of increasing MOI on uptake of M. tuberculosis. hMDMs were stimulated with FITC labeled γ-irr Mtb at different ratios and analyzed by flow cytometry. Data are presented as percentage of hMDMs that had phagocytosed at least one bacterium (FITC+). Values represent mean + SEM (n = 4). (TIF) [file pone.0101514.s002.tif]
